# Tracheostomy Care Knowledge among Clinical Nurses in Birat Medical College Teaching Hospital: An Observational Study

**DOI:** 10.31729/jnma.9117

**Published:** 2025-06-30

**Authors:** Bipin Koirala, Rajeev Kumar Shah, Rahul Mahato

**Affiliations:** 1 Department of ENT and HNS, Birat Medical College Teaching Hospital, Morang, Biratnagar

**Keywords:** *Intensive care units*, *knowledge*, *policy*, *tracheostomy care*

## Abstract

**Introduction:**

Tracheostomy, a surgically created opening in trachea, helps to maintain the airway in any respiratory problems. Its post-operative complications reflects the need of proper postoperative care. Good tracheostomy care knowledge helps early detection and timely management of tracheotomy related complications. Thus our study intends to assess the tracheostomy care knowledge among the clinical nurses in Birat Medical College Teaching Hospital.

**Methods:**

This cross-sectional observational study was conducted among nurses of ICU, high care, postoperative, and ENT wards at Birat Medical College Teaching Hospital from June to September 2024. A validated 20-item true/false questionnaire assessed tracheostomy care knowledge. Data were analyzed using SPSS v21 after ethical approval and informed consent.

**Results:**

Of 136 nurses, 50 (36.76%) had high knowledge, 40 (29.41%) moderate, and 46 (33.83%) low. High knowledge was found in 6 (100%) nurses with a master's degree, 35 (48%) working in ICU, and 6 (75%) with 10 to 20 years of experience. Correct responses ranged from 38 (27.94%) on tube reinsertion to 135 (99.26%) on securing tracheostomy ties.

**Conclusions:**

One-third of nurses had high tracheostomy care knowledge; higher scores were observed among ICU nurses and those with over ten years of experience.

## INTRODUCTION

Tracheostomy is a crucial intervention in managing airway obstruction, prolonged ventilation and patients with respiratory failure.^1^ It is a surgically created opening in the trachea creating an alternative route of respiration.^2^ Complications like tube blockage, displacement, and bleeding, accidental decannulation reflects the pivotal role of post-tracheostomy care. ^3^ Good bedside nursing can help many tracheostomy related complications by early detection and timely management. Complications related to tracheostomy which occurs in 30% of cases can be easily detected and handled with a correct approach.^4^ It has been seen that nurses working in wards are lacking in skills, knowledge and confidence to provide safe tracheostomy care.^5^ Such knowledge gaps reflects the need of systematic training as well as standardized protocol in improving patient's safety. Therefore, study intends to assess the tracheostomy care knowledge among the clinical nurses in Birat Medical College Teaching Hospital.

## METHODS

This observational cross-section study was performed among clinical nurses of Birat Medical College Teaching Hospital, Biratnagar, Nepal. Ethical approval was taken from the intuitional review committee of Birat Medical College Teaching Hospital (Reference number: IRC-PA386/2024).

A written informed consent was obtained from all participants before study. The study was conducted from 20 June 2024 to 20 September 2024. All nurses of Intensive Care Unit (ICU), high care, postoperative ward and Ear, Nose and Throat (ENT) ward willing to be the part of study were included in the study. Nursing staffs of other department were excluded from the study. Primary researcher personally visited respective wards for data collection.

The respondents were asked to fill a composite preformed 20 true and false questionnaire prepared in English language.^6^ Anonymity and confidentiality of data was maintained. Face validity was done among 20 nurses to identify ambiguous and confusing questions. Feedback confirmed that the questionnaire was clear and understandable. Each correct response was allotted a score of one with total 20 points. Score of less than 60% (Less than 12 score) was considered low, 60-80% (12-16) was considered moderate and more than 80% (>16) was considered to be of high knowledge.^7^ Data was recorded and analysis was done using SPSS (21 version).

## RESULTS

A total of 136 nurses were evaluated, out of which 73 worked in Intensive Care Unit (ICU) (Level II and Level III), 37 worked in high care (Level I), 18 in postoperative ward and eight in ENT ward. The mean age of nurses was 26.00±8.12 years. Seventy nurses had done Proficiency Certificate Level (PCL) nursing, 60 Bachelor level and six master level education. Among these 40 nurses had taken basic life support training.

Our analysis showed that 50 (36.76%) had high knowledge, 40 (29.41%) had moderate and 46(33.83%) had low knowledge about tracheostomy care (Table 1). However, the overall average score showed, moderate (70.6±20.2%) knowledge about the tracheostomy care among the clinical nurses.

**Table 1:** Overall tracheostomy care knowledge of clinical nurses in Birat Medical College Teaching Hospital (n=136).

Knowledge level	n(%)
High (>80%)	50(36.76)
Moderate	40 (29.41)
Low	46(33.83)

**Table 2:** Overall tracheostomy care knowledge of clinical nurses according to their training status, education and workplace (n=136).

Variables (n)	High n(%)	Mod n(%)	Low n(%)
Education level (n)			
Masters (6)	6(100.00)	0	0
Bachelors (60)	32(53.33)	20(33.33)	8(13.34)
PCL (70)	12(17.14)	20(28.57)	38(54.29)
Work Area (n)			
ICU (73)	35(48.00)	25(34.00)	13(18.00)
High care (37)	10(27.00)	20(54.00)	7(19.00)
Post operative ward (18)	3(17)	10(55)	5(28)
ENT ward (8)	2(25.00)	3(37.00)	3(37.00)

**Table 3:** Tracheostomy care knowledge by nursing experience.

Work experience(n)	Knowledge score(mean±SD)	Score distribution n(%)
< 5 years (80)	11.80±3.80	50 scored < 60%
		45 scored 60-80%
		5 scored >80%
5-10 years(48)	14.0±3.0	25 scored <60%
		60 scored 60-80%
		15 scored >80%
>10 years (8)	17.60±1.20	0 scored <60%
		25 scored 60-80%
		75 scored >80%

**Table 4:** Frequency of correct responses among nurses. (n=136)

Components	n(%)
Secretions around a tracheostomy cannula may irritate the skin	132(97.05)
A patient with a tracheostomy tube cannot be fed orally	85(62.50)
A spare tracheostomy tube should always be available due to the risk of accidental dislodgement	131(96.32)
A patient with a tracheostomy tube should receive regular oral hygiene	133(97.79)
Tracheostomy tubes are more efficient 73(53.67) for the aspiration of respiratory tract secretions	
Acute obstruction of a tracheostomy tube is most likely caused by a mucous plug	120(88.24)
Regular tracheostomy tube changes are always done by the beside nurse	54(39.71)
It is unimportant to keep a tracheostomy kit available at the bedside for emergency	90(66.17)
A patient with a dislodged tracheostomy 38(27.94) tube that cannot be inserted should be intubated with a endotracheal tube	
The pressure needed for tracheostomy tube auctioning is 180 - 200 mmHg	95(69.85)
A tracheostomy opening should always be kept dry and clean to prevent infection	134(98.53)
The tracheostomy cuff pressure should always be 40-50 mmHg	75(55.14)
The tracheostomy tie should be checked and secured regularly to avoid accidental dislodgement.	135(99.26)
It is unimportant to measure the cuff pressure regularly for a cuffed tracheostomy tube.	130(95.59)
Suctioning a tracheostomy tube must not take more than 10 seconds.	126(92.65)
For routine auctioning, the length of the suction catheter should exceed the length of the tracheostomy to be effective.	129(94.85)
Bleeding from the tracheostomy cannula is the most common complication.	128(94.12)
The dressing used for stomal infection is Aquacel Ag	131(96.32)
Patients require auctioning from the tracheostomy tube more frequently during the night rather than the day.	125(91.91)
Tracheostomy tie changes should be done daily.	133(97.79)

All nurses with master's degree had high knowledge compared to PCL level nurse where 38 (54.33%) had low knowledge. Thirty-five (48%) nurses working in ICU had high knowledge and 25 (34%) had moderate knowledge (Table 2).

The average knowledge score in nurses who had worked more than 10 years was 17.60±1.20% with 75 (75.00) scoring >80% (Table 3).

Out of all nurses included in the study 132 (97.05%) knew that secretions around a tracheostomy cannula may irritate the skin, 133 (97.79%) knew that a patient with a tracheostomy tube should receive regular oral hygiene and tracheostomy tie changes should be done daily(Table 4).

## DISCUSSION

There is a rise of tracheostomy procedure in recent days. Around 10-24% of ICU patients need tracheostomy.^7^ Although tracheostomy is performed by otolaryngologist or anesthesiologist, the postoperative caregivers are mainly the nursing staffs. Thus among various nursing cares, tracheostomy care had acquired more attention as several death has occurred as a result of nurses insufficient knowledge.^8,9^ It is thus very important to assess the knowledge and update on continuous professional development. Hence we conducted the study to evaluate the knowledge of clinical nurses about the tracheostomy care.

Our analysis showed that 50 (36.76%) had high knowledge, 40 (29.41%) had moderate and 46 (33.83%) had low knowledge about tracheostomy care. This bimodal knowledge level could be due to specialized training in specialized working area where tracheostomy care skills are practiced daily leading to high knowledge. Contrary to this study by Fahad et al showed that 58.7% the nurses had poor knowledge, followed by moderate in 39.00 % and least (2.31%) had high level of knowledge.^10,11^ Study by Qadir et al also showed similar results.^12^ This may be due the training variability and workload exposure in these studies as most them were ward nurses rather than ICU nurses.

However, the overall average score showed, moderate (70.6±20.2%) knowledge among the nurses. It indicates significant disparities in the knowledge, it suggests that more training and post educational classes should focus on the nurses scoring <60%. Similar results were seen in the study done by Gaterega et al and Dhaliwal et al.^13,14^ Study by Pradhan et al also reported 87.5% of respondents with good knowledge about the tracheostomy care.^15^ However, study by Mungan et al showed the knowledge of nurses were as low as 46.4%.^4^ Similarly, study by Padma et al also showed moderate knowledge in about 53.3% of the nurses. ^16^ In contrary to this Quadir showed 81.67% of nurses had inadequate knowledge.^12^

Most of the nurses were staff nurses 70 (51.47%) followed by nurses with Bachelor's degree 60 (44.12%) and least six (4.41%) had Master's degree. This result was same with Nwakaego et al where less than half had Bachelor's degree and minority were with Master's degree. ^9^ All nurses with a master's degree (100%) had high knowledge. This shows that advanced education correlates well with the critical thinking and evidence based practice. In contrast, only 12 (17.14%) of PCL nurses had high knowledge, and over half (54.29%) had low knowledge. This clearly reflects the higher education degree backs up the knowledge level including about the tracheostomy care. We know that the diploma courses usually emphasizes on task completion over conceptual understanding, thus it is necessary to bridge this gap focusing on more dedicated training. Rotational training of ward nurses to ICU may also help for better exposure ultimately better knowledge.

In our study nurses working in ICUs had the highest proportion with high knowledge 35(48.00%), compared to 10(27%) in high care, 3(17%) in post-operative wards and 2(25%) in ENT ward. This could be due to the fact that more trained and the ones with more education level are prioritized in ICU while less trained as with lesser education degree are allotted in the wards. Similarly ICU patients more commonly have tracheostomies due to prolong ventilation as well as neurologic injuries leading to higher clinical exposure seen in nurses working in ICU. Thus higher exposure with frequent hands on practices explains higher knowledge level. Similar to this study by Jamaldeen et al and Mugan et al also showed satisfactory knowledge in nurses working in ICU. ^3,4^

Nurses with >10 years' experience had the highest average knowledge score (17.60±1.20), and 75% scored above 80%. In contrast, those with <5 years' experience had the lowest average score (11.80±3.80) with the majority scoring <60%. Contrary to this in study by Sahyoun et al showed that nurses with work experience less than five years had highest level of knowledge. ^6^ That could be possibly due to up to date education, as compared to older professionals that have not been updated with the recent advances.

In the present study almost all nurses correctly identified the need for regular oral hygiene by 133(97.79%) nurses, importance of keeping the stoma clean and dry by 134 (98.53%), Daily tie changes by133 (97.79%) nurses, mucous plug as a cause of acute obstruction by 120 (88.24%) nurses, the importance of suctioning duration (<10 seconds) by126 (92.65%) nurses, importance of keeping spare tracheostomy tube and kit available by 131(96.32%)and 130 (95.59%),nurses respectively. The reinforcement of the hospital protocols like mandatory checklist with repetition of the performance and availability of the equipment might have improved the oral hygiene stoma care, daily tie changes and keeping the spare tracheostomy tube and kit. This also reflects the effective protocol dissemination as well. The knowledge about the mucous plug identification as obstructive cause clearly highlights the successful simulation in emergency preparedness of the clinical nurses.

However, only 38(27.94%) nurses knew that an endotracheal tube should be used when reinsertion of a dislodged tube fails, 73(53.67%) nurses recognized that tracheostomy tubes are more efficient for suctioning, 75(55.14%) nurses knew the correct cuff pressure (40-50 mmHg) and 90(66.17%) nurses falsely believed it's unimportant to keep a tracheostomy kit bedside.

Low exposure with reliability on doctors for endotracheal tube reinsertion after dislodgment might be the reason for lesser knowledge about it. Misconception about the technical details might also be the reason for the correct procedural requirement for tracheostomy and its care. The cuff pressure of 40-50 mm is outdated, this misconception shows requirement of updated knowledge in the nurses as well.

Many other studies focused on different components of the knowledge about the tracheostomy care. Study by Zeb et al showed 83% knew about the suctioning pressure and timing.^17^ Contrary to this, in the study done by Pinto et al only 46% of nurses were aware about the suctioning pressure. ^18^ Study by Kolethekkat et al most of the nurses were familiar about the tube and its function. ^19^ Study by Kolethekkat et al showed more than three forth of the respondents were aware about the stomal care. ^19^ Contrary to this, in study done by Azizan et al only 60% were aware about the safe fit of the tracheostomy tie.^20^ In study by Casserly et al only 45.7% were aware about the safe tie. ^21^ According to Dorton et al, significant deficiency in tracheostomy care is seen among primary health care providers. ^22^

The present study shows that the current protocols of our hospital is very strong in routine care mastery as well as emergency preparedness in the tracheostomy care but still there are gaps like persistence of outdated guidelines (eg cuff pressure) and also preparedness of rare scenarios, like cuff/ tube dislodgement knowledge, are being neglected. Thus it is very important to upgrade the knowledge especially by updated training programs to prevent complications related to tracheostomy care. Targeted courses as well stimulation labs would be beneficial. Improving the policy level in the hospital by mandating the tracheostomy competency certification especially for the dedicated wards would also be helpful.

Our study has few limitations. This study was done in single private hospital with limited sample size hence the findings may not be generalized. Also, this study has only assessed about the knowledge, so future studies should include about the attitude as well as practice of the tracheostomy care.

## CONCLUSIONS

This study revealed that the overall knowledge of nurses regarding tracheostomy care was suboptimal, with only one-third demonstrating high competency. Those working in intensive care settings and with more than ten years of experience showed better understanding. Although nurses showed good understanding of routine care elements like hygiene and suctioning, notable gaps persisted in critical emergency management areas, such as the proper response to tracheostomy tube dislodgement and maintaining correct cuff pressure.
